# A Biochemical Deconstruction-Based Strategy to Assist the Characterization of Bacterial Electric Conductive Filaments

**DOI:** 10.3390/ijms24087032

**Published:** 2023-04-11

**Authors:** Marta A. Silva, Ana P. Fernandes, David L. Turner, Carlos A. Salgueiro

**Affiliations:** 1 Associate Laboratory, i4HB—Institute for Health and Bioeconomy, School of Science and Technology, NOVA University Lisbon, 2819-516 Caparica, Portugal; 2 UCIBIO—Applied Molecular Biosciences Unit, Chemistry Department, School of Science and Technology, NOVA University Lisbon, 2829-516 Caparica, Portugal; 3 Instituto de Tecnologia Química e Biológica António Xavier, NOVA University Lisbon, 2780-157 Oeiras, Portugal

**Keywords:** *Geobacter*, nanowires, multiheme cytochromes, electron transfer, NMR

## Abstract

Periplasmic nanowires and electric conductive filaments made of the polymeric assembly of *c*-type cytochromes from *Geobacter sulfurreducens* bacterium are crucial for electron storage and/or extracellular electron transfer. The elucidation of the redox properties of each heme is fundamental to the understanding of the electron transfer mechanisms in these systems, which first requires the specific assignment of the heme NMR signals. The high number of hemes and the molecular weight of the nanowires dramatically decrease the spectral resolution and make this assignment extremely complex or unattainable. The nanowire cytochrome GSU1996 (~42 kDa) is composed of four domains (A to D) each containing three *c*-type heme groups. In this work, the individual domains (A to D), bi-domains (AB, CD) and full-length nanowire were separately produced at natural abundance. Sufficient protein expression was obtained for domains C (~11 kDa/three hemes) and D (~10 kDa/three hemes), as well as for bi-domain CD (~21 kDa/six hemes). Using 2D-NMR experiments, the assignment of the heme proton NMR signals for domains C and D was obtained and then used to guide the assignment of the corresponding signals in the hexaheme bi-domain CD. This new biochemical deconstruction-based procedure, using nanowire GSU1996 as a model, establishes a new strategy to functionally characterize large multiheme cytochromes.

## 1. Introduction

The electrogenic *Geobacter* bacteria are predominant in several natural habitats due to their high respiratory versatility, including the use of either intracellular or extracellular terminal electron acceptors to generate the necessary metabolic energy from the degradation of organic compounds [1,2,3]. The coupling of the oxidation compounds to the reduction of terminal intracellular acceptors is transversal in the entire bacteria kingdom and the mechanisms are generally well understood. In contrast, the electron transfer mechanisms adopted by electrogenic bacteria to couple their respiratory chains to the use of extracellular compounds are still under intense research. This is the case of the bacterium *Geobacter sulfurreducens*, which can couple the oxidation of organic compounds to the reduction of insoluble metal oxides, some of them pollutants and radioactive [4,5]. In addition, the bacterium is also capable of transferring electrons to electrode surfaces from which electricity can be harvested [6]. Overall, these capabilities have led to an increasing interest towards the application of *Geobacter* cells in the fields of bioenergy and bioremediation [3,7,8,9,10]. The foundations for the metabolic versatility shown by *G. sulfurreducens* results from the intricate electron transfer network involving *c*-type cytochromes, most of them containing multiple heme groups. Indeed, these cytochromes are found at the inner membrane, periplasm and at the outer membrane (for a review see [11]). Recently, cryogenic electron microscopy (Cryo-EM) has revealed that *G. sulfurreducens* can also form electrically conductive filaments made of the polymeric assembly of multiheme cytochromes, as it is the case of the tetraheme OmcE [12], the hexaheme OmcS [13] and the octaheme OmcZ [14].

Contrasting with the polymeric assembly of mature cytochromes, the presence of which cannot be anticipated by genomic analysis, *Geobacter* cells encode for monomeric periplasmic *c*-type cytochromes with unusual high numbers of hemes, previously designated as nanowires of hemes [15]. Three were found in the *G. sulfurreducens* genome—GSU2210 (with 27 hemes), GSU0592 and GSU1996 (both with 12 hemes) [16]. Homologous proteins of the last two cytochromes were also found in the genome of *G. metallireducens* and *G. uraniireducens* [16,17]. The role of these multiheme cytochromes and their location at the periplasm indicated that they were designed to function differently from the bacterial conductive filaments made of the polymeric assembly of cytochromes OmcS, OmcE or OmcZ, which extend from the periplasm to outside the cell, thus permitting the long-range electron transfer to extracellular acceptors and their concomitant reduction [12,13,14]. Instead, periplasmic nanowires most likely function as bacterial electron-storage devices or capacitors [18,19]. These electron reservoirs are intimately associated with the respiratory versatility of *Geobacter* cells, since continuous production of energy is warranted even when extracellular electron acceptors are exhausted, thus providing enough survival time for the bacteria to find new and appropriate environments [18].

**Figure 1:**
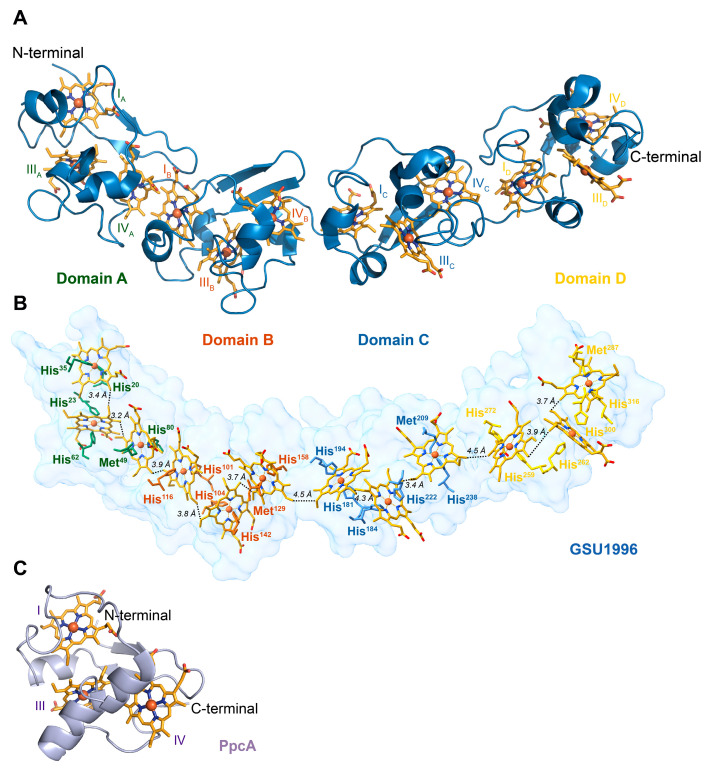
Structure of the cytochrome GSU1996 (PDB code 3OV0 [15]) and the triheme cytochrome PpcA (PDB code 2LDO [21]) from *Geobacter sulfurreducens*. In the structures, the heme groups are colored orange. Hemes are numbered I, III and IV according to the order of their attachment to the CXXCH motifs in the polypeptide chain to maintain consistency with the literature [15,22]. (**A**) Structure of cytochrome GSU1996 highlighting each triheme domain (A–D). (**B**) Surface representation of cytochrome GSU1996 showing the heme axial ligands and the heme array with the minimum observed distances indicated between adjacent porphyrin rings. (**C**) Structure of triheme cytochrome PpcA.

The structure of the monomeric dodecaheme cytochrome GSU1996 (42 kDa) has been determined by X-ray crystallography [15]. The hemes have a linear arrangement along a single polypeptide chain in a “crescent shape” (Figure 1). The hemes are packed as a wire with edge-to-edge distances between 3 and 5 Å, which facilitates the electron tunneling between the cofactors, assuring a proper electron transfer [20]. The protein is organized in four domains (named A, B, C and D), each containing three heme groups connected by short linker regions. The triheme domains share high sequence homology and are structurally comparable to periplasmic triheme *c*-type cytochromes [15,21]. However, while in the latter, all the hemes are axially coordinated by two histidine residues; in the dodecaheme cytochrome, each domain has one heme with His-Met coordination.

Lengthy cytochromes containing many heme groups are unquestionably important either for long-range electron transfer, as is the case of the bacterial electric conductive filaments OmcE, OmcS and OmcZ, or to function as capacitors as in the case of cytochrome GSU1996. These properties can be conceivably explored for practical biotechnological applications. Yet, this would require detailed knowledge of the functional proprieties of these nanowires.

The functional characterization of multiheme cytochromes, including the determination of the heme’s reduction potential values, their modulation by the oxidation state of their neighbors (redox-interactions), as well as its modulation by the pH (redox-Bohr interactions) cannot be assessed by simple potentiometric or voltametric redox titrations. In fact, such approaches fail in discriminating the individual hemes and do not provide the necessary mechanistic information to properly describe the functional behavior of the redox centers in multiheme cytochromes (for a review see [23]). This can be attainable by NMR spectroscopy, since each heme has a distinct set of signals, which are also quite different in reduced and oxidized states. Thus, the signals of each heme, namely those of the heme methyls, can be independently traced from their initial position in the reduced state to their final oxidized state using 2D-exchange spectroscopy (EXSY) to monitor the stepwise oxidation of the different hemes in multiheme cytochromes (for a review see [23,24]). The chemical shift of the heme signals, in the different oxidation stages, depends upon the hemes’ relative microscopic reduction potential values, thus providing information on their oxidation profiles [25,26].

To distinguish the individual oxidation profiles of the hemes, it is first necessary to assign the heme proton signals. The most cost-effective manner to assign these signals encompasses the use of natural abundance samples. In these samples, it is usually sufficient to combine the information obtained from 2D ^1^H-nuclear Overhauser effect spectroscopy (NOESY) and 2D ^1^H-total correlation spectroscopy (TOCSY) or 2D ^1^H correlation spectroscopy (COSY) for samples in the reduced state [27] or by combining these experiments with 2D ^1^H,^13^C heteronuclear multiple quantum coherence (HMQC) for samples in the oxidized state [28]. In this state, the heme protons’ signals are differently affected by the orientation of the magnetic axes of the paramagnetic heme(s) and, hence, are highly variable even within a highly homologous groups of proteins [29]. Although larger signal dispersion is observed for oxidized samples, the assignment of the heme proton signals in a reduced form is more straightforward since they are essentially determined by the heme ring-current effects and can be found in very typical spectral regions [27,30]. There have been several possible strategies to assign heme signals described in the literature [27,31,32,33]. The seminal work of Wütrich’s group [31] described the assignment of heme protons in the reduced form of the monoheme horse heart cytochrome *c*. The same approach was later implemented to assign these signals in a tetraheme cytochrome *c*_3_ from *Desulfovbrio vulgaris* [27,28]. Since then, and until today, this strategy has been successfully used to assign the heme substituents from many multiheme cytochromes in their natural abundance state (for a review see [24,34]). Alternative methodologies using ^13^C-enriched porphyrin samples have also been described in the literature to assist in the assignment of some heme substituents in monoheme cytochromes or to obtain diverse structural information [32,33].

**Figure 2:**
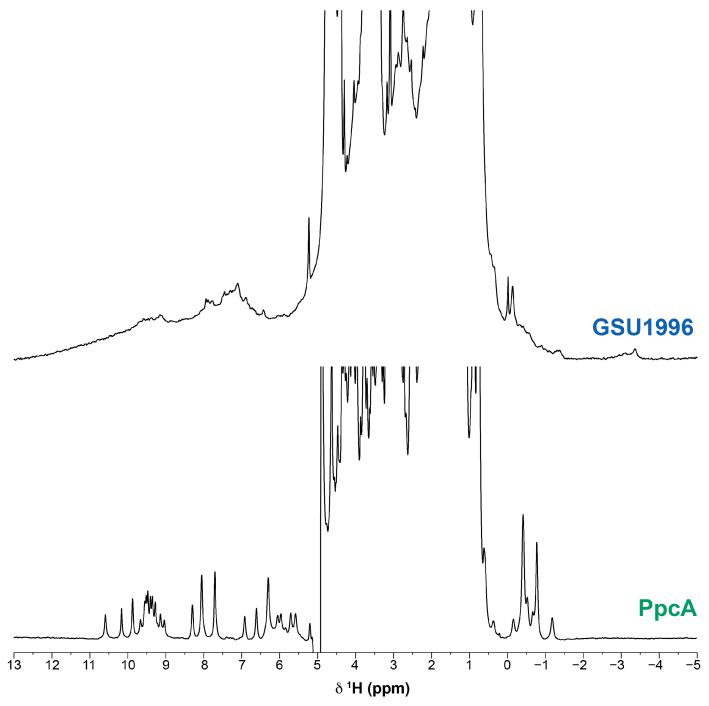
1D ^1^H-NMR spectra of cytochrome GSU1996 (upper) and triheme cytochrome PpcA (lower) from *Geobacter sulfurreducens* in the reduced state (pH 8.0 and 288 K). The spectra were recorded on a spectrometer operating at a proton frequency of 600 MHz.

The high number of heme groups and the concomitant high molecular weight of nanowire cytochromes considerably decrease the NMR spectral quality due to signal broadness. Consequently, the necessary assignment of the heme proton signals is compromised, as it is the subsequent strategy necessary for the characterization of the proteins’ functional mechanism. To illustrate the effect of the increase in the molecular weight on the spectra quality, the 1D ^1^H-NMR spectra for the dodecaheme cytochrome GSU1996 (~42 kDa) and triheme cytochrome PpcA (~10 kDa) are represented in Figure 2. In fact, the considerable signal broadening observed for the signals of cytochrome GSU1996, as well as the number of heme proton signals (114, excluding the heme propionate groups) would impair their completefull and unequivocal assignment.

Given the potential of nanowire cytochromes to be explored as capacitors, key components for bioenergy production and even to act as scaffolds for the next generation of biogenic electronic nanomaterials, efforts need to be undertaken to functionally characterize these biological systems. The modular deconstruction of nanowires of hemes and electric conductive bacterial filaments can conceivably be explored to assist in their characterization. As mentioned above, the number of hemes and the molecular weight of the full-length proteins will often impair their detailed functional characterization. Indeed, such characterization has been, to date, limited to cytochromes containing four heme groups. In the case of bacterial filaments, efforts should be directed toward the expression and biochemical characterization of their monomers. On the other hand, for monomeric nanowires of hemes, the expression of individual domains, for which characterization is relatively straightforward, followed by the characterization of different combinations of these domains, is a feasible strategy.

In the present work, we used the monomeric dodecaheme cytochrome GSU1996 as a model to illustrate a strategy that can be explored in the future to characterize nanowire cytochromes at the microscopic level. We used a deconstruction-based biochemical approach in which the triheme domains (A–D) and hexaheme bi-domains (AB and CD) of the dodecahemic protein were independently produced. Enough protein was obtained for the individual domains C/D and bi-domain CD, which were then used to illustrate the methodology developed in this work. The successful assignment of the heme proton signals in the triheme domains C and D were then used as a guide to assign the signals from the hexaheme bi-domain CD. The presented strategy paves the way for a future characterization of the electron transfer mechanisms in periplasmic nanowires and electric conductive bacterial filaments.

## 2. Results and Discussion

The cytochrome GSU1996 and respective domains were expressed and purified as previously described in the literature (see Section 3).

**Figure 3:**
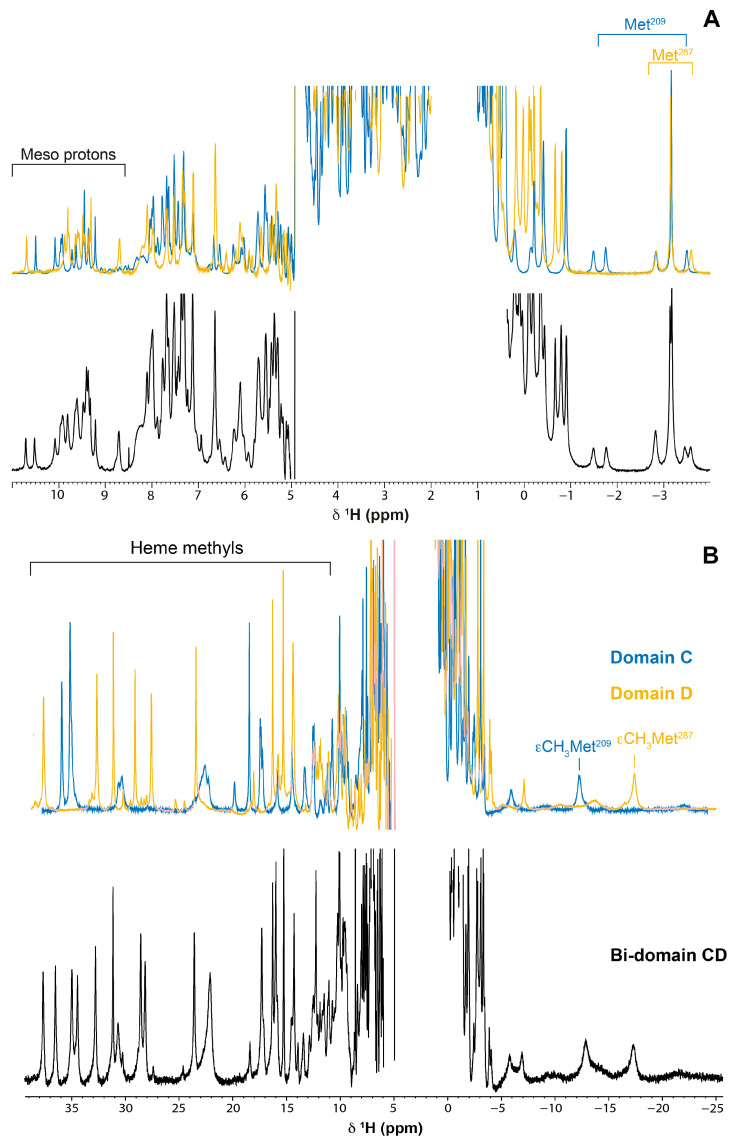
1D ^1^H-NMR spectra of domain C, domain D and bi-domain CD in the reduced (**A**) and oxidized state (**B**). The spectra of domains C and D are represented in the top of each panel in blue and yellow, respectively. The bottom spectrum in each panel correspond to bi-domain CD. The typical regions for meso and methyl signals are indicated in the reduced and oxidized spectra, respectively. The axial methionine spectral signatures are indicated in the reduced spectra of domains C and D. The signal correspondent to the group of εCH_3_ of each axial methionine is also indicated in the oxidized spectra. The spectra were recorded at pH 8 and 288 K on a spectrometer operating at a proton frequency of 600 MHz.

The crucial step underlying the characterization of the functional mechanisms of multiheme cytochromes requires the assignment of the heme protonsto the specific hemes in the structure, namely the methyl groups. The 1D ^1^H-NMR spectra of domains C, D and bi-domain CD are characteristic of low-spin *c*-type cytochromes in both the reduced and oxidized states (Figure 3). The NMR signals cover the regions from −4 to 11 ppm and −20 to 40 ppm in the reduced and oxidized states, respectively. The pattern and linewidths of the NMR signals are clearly distinct from those of high-spin cytochromes [28]. In the latter, the 1D ^1^H-NMR spectra show extremely broad signals above 40 ppm in the oxidized state, while in the reduced form the signals cover spectral regions typically between −15 and 30 ppm. Such profiles are not observable for the triheme domains and hexaheme bi-domain CD, indicating that all hemes are diamagnetic (Fe(II), S = 0) and paramagnetic (Fe(III), S = 1/2) in the reduced and oxidized forms, respectively.

Analysis of the more shifted signals in either the reduced or oxidized spectra of the three proteins show that the NMR signals in the hexaheme bi-domain CD closely follow the spectral distribution of each individual domain (*cf*. top and bottom panels in Figure 3). Particularly, there is a remarkable similarity of the heme axial methionine’s side chain protons (Met^209^ and Met^287^ in domains C and D, respectively). The pattern for the NMR signals of axial methionine typically has a three-proton intensity peak at approximately −3 ppm and up to four one-proton intensity peaks in the same spectral region [35,36]. This pattern is observable in both triheme domains, as well as in the hexaheme bi-domain where both sets of signals are clearly observable (Figure 3A). Moreover, in the spectra of the bi-domain, the chemical shifts of the signals are remarkably similar to those of the individual domains, indicating that the heme core and the geometry of the heme axial ligands are conserved (Figure 3A).

Given the conserved geometry of the heme core, the assignment of the heme signals in each triheme domain can, conceivably, be used to assist their assignment in the hexaheme domain. To test this hypothesis, we then moved to the assignment of the heme proton substituents in the reduced proteins. The reason for this choice is based on two facts: (i) the diamagnetic state of the hemes—ensuring that less signal broadening will be observed when the two domains are studied as a whole; and (ii) the very well-defined spectral regions covered by the heme signals according to their type—the only exception is the propionate groups, since they are more structurally variable.

### 2.1. Assignment of the Heme Proton’s NMR Signals of Domain D in the Reduced State

The NMR heme proton signals of domain C in the reduced state were previously assigned and are not discussed here (for a review see [22]). The data obtained for this domain are only reported for the sake of completeness.

**Figure 4:**
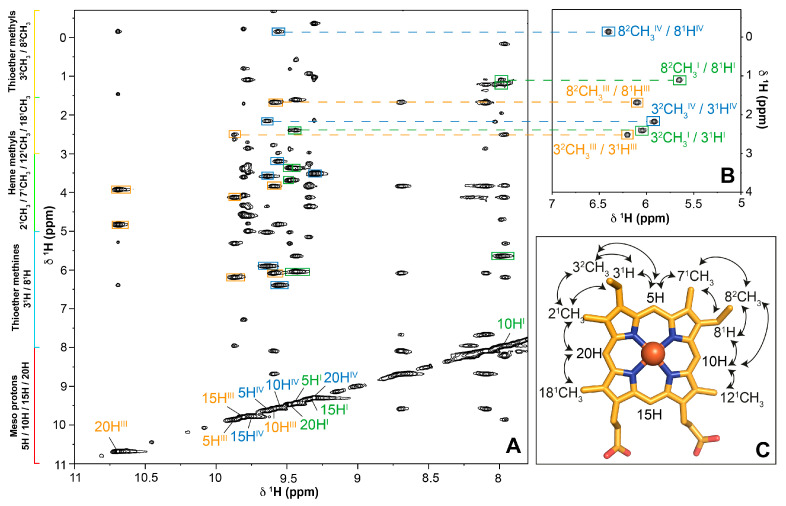
Illustration of the methodology used to assign the heme proton’s signals of domain D in the reduced state. (**A**) Expansion of a 2D ^1^H-NOESY NMR spectrum (80 ms mixing-time) region intraheme NOE connectivities for the heme meso protons. The typical regions for the heme proton signals are highlighted on the left of the spectrum; (**B**) Expansion of the 2D ^1^H-TOCSY NMR spectrum (50 ms mixing-time) highlighting the thioether methine/thioether methyls scalar coupling. The dashed lines connect the thioether methyl’s signals with the corresponding meso proton in the 2D ^1^H-NOESY NMR spectrum; (**C**) Diagram of heme *c* numbered according to the IUPAC-IUB nomenclature [46]. The full lines represent the interheme NOE connectivities. The spectra were recorded at pH 8 and 288 K on a spectrometer operating at a proton frequency of 600 MHz.

In the present work, we assign the heme proton’s signals of domain D using the strategy previously described for multiheme cytochromes [27]. Briefly, this strategy explores the typical regions covered by the heme signals: 11–8 ppm for meso protons (5H, 10H, 15H and 20H); 8–6 ppm for thioether methine (3^1^H and 8^1^H); 5–2.5 ppm for heme methyls (2^1^CH_3_, 7^1^CH_3_, 12^1^CH_3_ and 18^1^CH_3_) and 3–(−1.0) ppm for thiother methyls (3^2^CH_3_ and 8^2^CH_3_) (Figure 4A) [27,37,38,39,40,41,42,43,44,45]. Amongst all these substituents, only the thioether methine/thioether methyls (3^1^H/3^2^CH_3_ and 8^1^H/8^2^CH_3_) groups are scalar coupled. Thus, the first step of the assignment encompasses the identification of these pairs in the 2D ^1^H-TOCSY NMR spectra (Figure 4B). Then, the distinctive pattern of short-range intraheme connectivities established between meso protons and their neighboring substituents is identified in the 2D ^1^H-NOESY NMR spectra acquired with short mixing-times (50–100 ms). Meso protons 20H are only connected to two heme methyls (2^1^CH_3_ and 18^1^CH_3_) and meso protons 15H show no connections to heme methyls or thioether substituents (see arrows in Figure 4C). The meso protons 5H and 10H are both connected to one heme methyl, one thioether methine and one thioether methyl yielding the same pattern of NOE connectivities. The distinction between these meso protons is obtained by the inspection of their connectivities with heme groups 2^1^CH_3_/3^2^CH_3_ and 7^1^CH_3_/8^2^CH_3_. The short-range intraheme NOE connectivities for the meso protons are indicated in Figure 4A.

Two-dimensional ^1^H-NOESY experiments performed with a mixing time in the range of 150–400 ms allowed for the observation of the long-range intraheme connectivities and further confirmed the assignment of the signals. It is worth mentioning that the mixing-time ranges should be considered as a guide and it is recommended that, in absence of previous knowledge of the systems, NOE buildup curves to assist the selection of the mixing time should be investigated. However, triheme cytochromes have been extensively studied by our group, including the abovementioned domain C (for a review see [34]) and the values of the mixing times are well optimized to exclude possible bias caused by spin diffusion. In the present work, 2D ^1^H-NOESY were acquired with 80 and 200 ms mixing times. The heme proton chemical shifts of domains C and D in the reduced state are listed in Table 1.

### 2.2. Cross-Assignment of the Hemes to the Structure of Domain D

**Figure 5:**
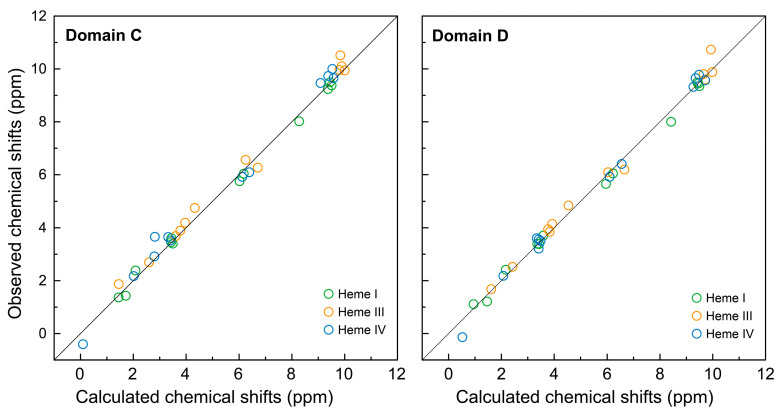
Comparison of observed and calculated chemical shifts of the heme protons from domains C and D. The calculated values were obtained from the crystal structure of the bi-domain CD (PDB code 3OUE [15]). The solid line has a unit slope. The RMSD values of the hemes I, III and IV are, respectively, 0.02, 0.05 and 0.07 for domain C and 0.02, 0.04 and 0.04 for domain D.

After assigning the heme signals, we then moved to their specific assignment in the protein structure. To achieve this, the observed chemical shifts were compared to those calculated from the crystal structure of bi-domain CD (Figure 5 and Table 2).

Of the six possible permutations for the three sets of heme protons with respect to the crystal structure, one was clearly preferred since all hemes concurrently showed the smallest root mean square deviation (RMSD). The RMSD of the 36 shifts was 0.10 ppm, with deviations of 0.02 (heme I), 0.04 (heme III) and 0.04 (heme IV). As in the case of domain C [21], the observed and predicted shifts for domain D correlate very well, even for the protons subjected to the larger ring current effects, as it is the case of the protons 10H^I^, 20H^III^, 2^1^CH_3_^III^, 12^1^CH_3_^I^, 8^2^CH_3_^I^ and 8^2^CH_3_^IV^ (*cf*. Figure 5 and Table 1).

The assignment of domain D heme signals was further tested by examination of the interheme NOE connectivities and their comparison with the distances obtained from the crystal structure of bi-domain CD. All NOE connectivities between protons up to 3 Å were observed in the 2D ^1^H-NOESY spectra, which confirms that both crystal and solution structures are similar.

### 2.3. Assignment of the Heme Signals of Bi-Domain CD in the Reduced State

**Figure 6:**
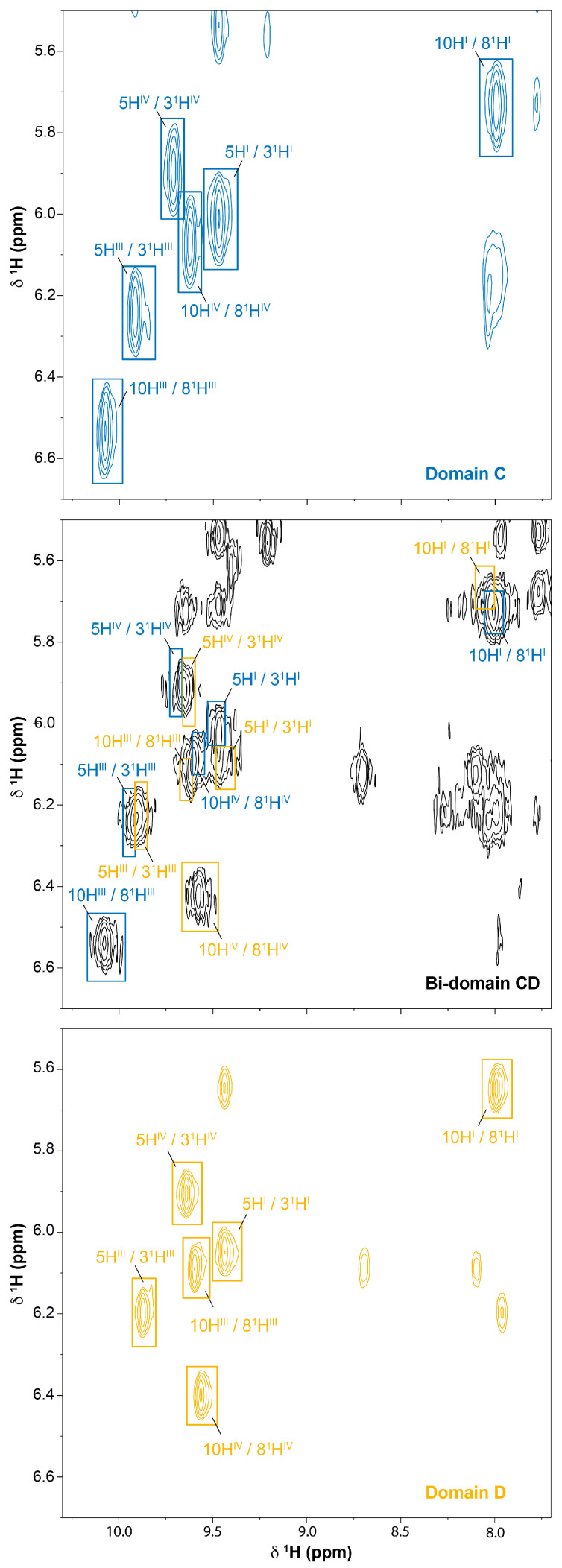
Expansion of a 2D ^1^H-NOESY NMR spectra region of domain C (blue), domain D (yellow) and bi-domain CD (black) highlighting the meso/thioether methine NOE connectivities (pH 8 and 288 K). All spectra were acquired with 80 ms mixing-time. The spectra were recorded on a spectrometer operating at a proton frequency of 600 MHz.

Compared to triheme domains, the bi-domain CD has twice the heme groups and molecular weight. Consequently, the bi-domain molecules have a longer correlation time, yielding broader signals compared to those of the individual domains. The higher number of heme signals and their broadness impairs the application of the assignment methodology described above for the individual domain D. However, as illustrated in Figure 3, the NMR spectra of the hexaheme bi-domain essentially corresponds to the superimposition of the spectra of each individual domain. Thus, in the present work, the assigned heme signals of domains C and D were used as a guide to assign the corresponding ones in the NMR spectrum of the bi-domain CD (Figure 6). As expected, the signals are broader and less dispersed in the 2D ^1^H-NOESY spectra of the bi-domain (*cf*. the three panels in Figure 6). This clearly indicates that, in the absence of an independent assignment of the signals in domains C and D, their ab initio assignment in the bi-domain would be unlikely. The heme protons assignment of the bi-domain CD is reported in Table 1.

**Figure 7:**
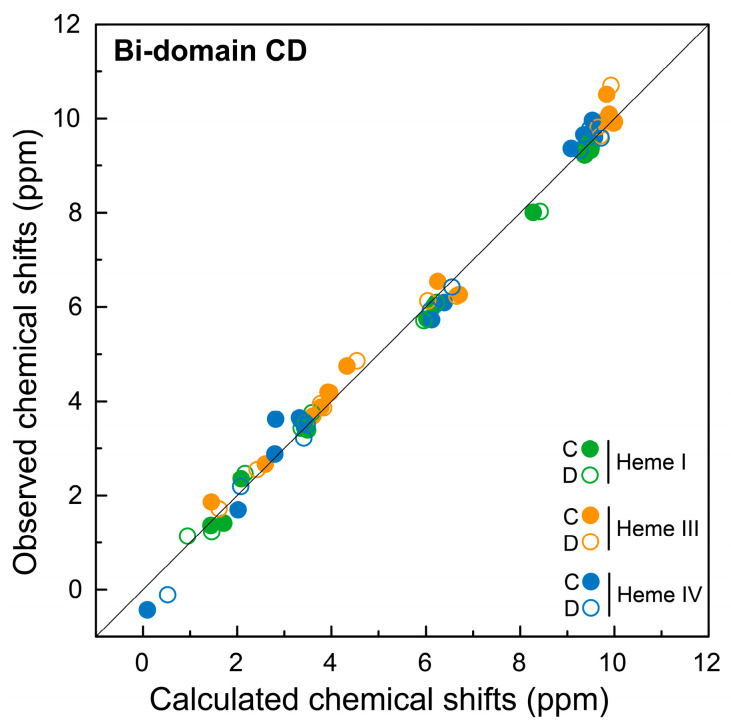
Comparison of the observed and calculated chemical shifts of the heme protons from hexaheme bi-domain CD. The calculated values were obtained from the crystal structure of the bi-domain CD (PDB code 3OUE [15]). The heme signals corresponding to domains C and D in the bi-domain CD are represented by filled and open circles, respectively. The solid line has a unit slope. The RMSD values of the hemes I, III and IV are, respectively, 0.02, 0.05 and 0.07 for domain C and 0.02, 0.04 and 0.03 for domain D.

As for the individual domains, the assignment of the bi-domain’s heme signals was confirmed by comparing the observed and the predicted chemical shifts (Figure 7 and Table 2). Furthermore, in the case of the bi-domain CD, the chemical shifts correlate very well, even for the protons with large ring current shifts. The assignment was further confirmed by the analysis of the interheme NOE connectivities expected from the analysis of the crystal structure. All the expected connectivities between the closest protons were observed in the 2D ^1^H-NOESY spectra and contributed to the validation of the strategy used in the present work to assign the heme signals in proteins containing many heme groups.

## 3. Materials and Methods

### 3.1. Expression and Purification of Proteins

The full-length cytochrome GSU1996, each triheme domain (A–D) and the hexaheme bi-domains AB and CD were expressed and purified as previously described [16,47,48,49]. The domains A–D and CD were expressed in *E. coli* strain JCB7123 [50], while AB and full-length cytochrome were produced in *E. coli* strain BL21 (DE3) [48]. Briefly, both *E. coli* strains harbor the plasmid pEC86 containing the *c*-type cytochrome maturation gene cluster *ccmABCDEFGH* [51]. The strains were aerobically grown, at 30 °C and 200 rpm, to mid-exponential phase and induced with 10 μM isopropyl β-D-1-thiogalactopyranoside (IPTG), except for bi-domain AB for which no induction was necessary. After overnight incubation, the cells were harvested and the periplasmic fraction was isolated by osmotic shock in the presence of lysozyme. The periplasmic fractions were dialyzed against 20 mM Tris-HCl buffer pH 8.5 (domain A), 10 mM Tris-HCl buffer pH 7.0 (domains C and D) or 20 mM sodium phosphate buffer pH 5.9 (bi-domains AB/CD and full-length protein) and loaded onto a cation-exchange column (Econo-Pac High S, Bio-Rad, CA, USA). The fractions were then eluted with a linear gradient of NaCl.

In each case, the fractions with the protein of interest were pooled, concentrated and loaded onto a HiLoad 16/600 Superdex 75 column (UK, Amersham, GE Healthcare), equilibrated with 20 mM sodium phosphate buffer pH 8.0 with 100 mM NaCl. The presence of the desired proteins was confirmed by 12% sodium dodecyl sulfate polyacrylamide gel electrophoresis (SDS-PAGE) stained with Coomassie blue. Both chromatography steps were performed in an ÄKTA Prime Plus FPLC System (UK, Amersham, GE Healthcare).

The triheme domains C and D, as well as the correspondent hexaheme bi-domain CD, showed the highest levels of expression. On the other hand, domain B was poorly expressed. For this reason, the triheme domains C/D and the hexaheme bi-domain CD were used to illustrate and validate the deconstruction-based biochemical strategy.

### 3.2. Sample Preparation for NMR Studies

The buffer used in the last step of the purification was exchanged for 80 mM sodium phosphate buffer pH 8.0 (with NaCl added to a final ionic strength of 250 mM) in 99.9% ^2^H_2_O (CIL), through ultrafiltration procedures with Amicon Ultra Centrifugal Filter Units (Millipore). Protein concentrations were determined by UV-visible spectroscopy with the specific absorption coefficient of the α-band at 552 nm determined for the reduced triheme cytochrome PpcA [22,52]. Protein samples with approximately 1.5 mM were placed in 3-mm Wilmad NMR tubes and closed with NMR pressure caps. The protein samples were degassed with H_2_ and reduced in the presence of catalytic amounts of Fe-hydrogenase from *Desulfovibrio vulgaris* (Hildenborough) [22].

### 3.3. NMR Experiments

The NMR spectra were acquired on a Bruker Avance 600 MHz spectrometer at 288 K. To assist the assignment of the heme proton signals of domains C, D and CD in the reduced state, a series of 2D ^1^H-TOCSY and 2D ^1^H-NOESY NMR spectra were recorded with standard pulse techniques and with mixing times of 50 and 80/200 ms, respectively. The spectra were acquired with 4096 (*t*_2_) × 512 (*t*_1_) data points, with 256 scans per increment, covering a sweep width of 20 kHz. The ^1^H chemical shifts are reference to sodium dodecyl sulfate (DSS) at 0 ppm, by using the residual water signal as an internal secondary reference [53]. All NMR spectra were processed with TopSpin 3.5.7^TM^ software (Bruker Biospin, Karlsruhe, Germany) and analyzed with Sparky (T. D. Goddard and D. G. Kneller, Sparky 3, University of California, San Francisco, CA, USA).

### 3.4. Calculation of Ring-Current Shifts

The ring-current shifts were calculated from the crystal structure of bi-domain CD [15] following the procedure described by Turner and co-workers [27]. The heme substituent chemical shifts were calculated through a correction of the heme protons reference shifts (9.36 ppm for meso protons, 6.13 for thioether methines, 3.48 for methyls and 2.12 for thioether methyls), as described by Pessanha and co-workers [37].

## 4. Conclusions

Nanowires of hemes and the polymeric assembly of *c*-type cytochromes forming electric conductive filaments are involved in extracellular electron transfer pathways in which they can transfer electrons to long range distances to outside of the cells or act as cellular capacitors. While structural models for these full-length proteins have been successfully obtained by X-ray crystallography, or more recently by Cryo-EM, the determination of their heme redox properties and the concomitant deciphering of their mechanistic and functional properties remains elusive.

The main reason for the lack of precise functional mechanistic information is explained by the high molecular weight and number of *c*-type heme groups. To date, detailed thermodynamic characterization, and hence, mechanistic information, have only been obtained for multiheme cytochromes containing up to four heme groups [54]. The same type of information must be obtained for the full-length nanowires of hemes or electric conductive bacterial filaments. The present work provides a first contribution towards this goal. Using the dodecaheme cytochrome GSU1996 as a model, containing four triheme domains (A to D), we present a strategy to assist the detailed characterization of larger multiheme cytochromes. This strategy encompasses the production at natural abundance of smaller individual triheme and hexaheme domains. We assigned the heme proton signals of the two C-terminal triheme domains (C and D) and used this assignment as a guide to assign the correspondent signals in the hexaheme bi-domain (CD). Future work must focus on the assignment of the heme proton signals for the two N-terminal triheme domains and the respective bi-domain. This would then allow for the monitorization of the oxidation profile of each heme in the full-length protein—as a ground to determine their redox properties—using the well-established methodologies for multiheme cytochromes [23].

The exemplified deconstruction-based strategy provides an effective tool by which to study the redox properties of the individual hemes in nanowires or electrically conductive filaments. In the case of nanowires of hemes with all redox centers connected by a unique polypeptide chain, as it is in the case of GSU1996, the expression of individual domains is an appropriate approach. In the case of electrically conductive bacterial filaments made of the polymeric assembly of cytochromes, as in the case of OmcE, OmcS and OmcZ filaments, the expression and characterization of their individual monomers can be similarly attained.

## Figures and Tables

**Table 1:** Observed ^1^H and ring current chemical shifts (in parentheses) of the heme protons of domains C, D and bi-domain CD in the reduced state at pH 8.0 and 288 K. Data for domain C were previously determined [22] and are indicated for completeness.

			Chemical Shifts (ppm)		
Heme Substituent	Domain		Heme I	Heme III	Heme IV
5H	C		9.49 (−0.13)	9.94 (−0.58)	9.73 (−0.37)
	D		9.44 (−0.08)	9.87 (−0.51)	9.64 (−0.28)
	CD	C	9.47 (−0.11)	9.93 (−0.57)	9.65 (−0.29)
		D	9.47 (−0.11)	9.89 (−0.53)	9.65 (−0.29)
10H	C		8.01 (1.35)	10.09 (−0.73)	9.64 (−0.28)
	D		7.99 (1.37)	9.59 (−0.23)	9.56 (−0.20)
	CD	C	8.00 (1.36)	10.08 (−0.72)	9.60 (−0.24)
		D	8.02 (1.34)	9.63 (−0.27)	9.58 (−0.22)
15H	C		9.37 (−0.01)	9.96 (−0.60)	9.99 (−0.63)
	D		9.34 (0.02)	9.80 (−0.44)	9.77 (−0.41)
	CD	C	9.36 (0.00)	9.96 (−0.60)	9.96 (−0.60)
		D	9.32 (0.04)	9.81 (−0.45)	9.78 (−0.42)
20H	C		9.23 (0.13)	10.51 (−1.15)	9.46 (−0.10)
	D		9.48 (−0.12)	10.69 (−1.33)	9.31 (0.05)
	CD	C	9.21 (0.15)	10.51 (−1.15)	9.36 (0.00)
		D	9.40 (−0.04)	10.70 (−1.34)	9.32 (0.04)
2^1^CH_3_	C		3.59 (−0.11)	4.74 (−1.26)	3.65 (−0.17)
	D		3.69 (−0.21)	4.83 (−1.35)	3.55 (−0.07)
	CD	C	3.56 (−0.08)	4.74 (−1.26)	3.61 (−0.13)
		D	3.75 (−0.27)	4.85 (−1.37)	3.55 (−0.07)
7^1^CH_3_	C		3.45 (0.03)	4.18 (−0.70)	3.64 (−0.16)
	D		3.38 (0.10)	4.13 (−0.65)	3.59 (−0.11)
	CD	C	3.43 (0.05)	4.17 (−0.69)	3.64 (−0.16)
		D	3.42 (0.06)	4.18 (−0.70)	3.60 (−0.12)
12^1^CH_3_	C		1.42 (2.06)	3.88 (−0.40)	2.91 (0.57)
	D		1.20 (2.28)	3.84 (−0.36)	3.20 (0.28)
	CD	C	1.40 (2.08)	3.87 (−0.39)	2.87 (0.61)
		D	1.22 (2.26)	3.85 (−0.37)	3.21 (0.27)
18^1^CH_3_	C		3.39 (0.09)	3.68 (−0.20)	3.51 (−0.03)
	D		3.37 (0.11)	3.93 (−0.45)	3.49 (−0.01)
	CD	C	3.38 (0.10)	3.68 (−0.20)	3.51 (−0.03)
		D	3.55 (−0.07)	3.94 (−0.46)	3.49 (−0.01)
3^1^H	C		6.03 (0.10)	6.26 (−0.13)	5.91 (0.22)
	D		6.04 (0.09)	6.19 (−0.06)	5.91 (0.22)
	CD	C	6.02 (0.11)	6.25 (−0.12)	5.72 (0.41)
		D	6.09 (0.04)	6.22 (−0.09)	5.92 (0.21)
8^1^H	C		5.75 (0.38)	6.55 (−0.42)	6.09 (0.04)
	D		5.65 (0.48)	6.09 (0.04)	6.40 (−0.27)
	CD	C	5.74 (0.39)	6.54 (−0.41)	6.09 (0.04)
		D	5.70 (0.43)	6.12 (0.01)	6.42 (−0.29)
3^2^CH_3_	C		2.37 (−0.25)	2.69 (−0.57)	2.16 (−0.04)
	D		2.40 (−0.28)	2.51 (−0.39)	2.17 (−0.05)
	CD	C	2.35 (−0.23)	2.66 (−0.54)	1.69 (0.43)
		D	2.46 (−0.34)	2.54 (−0.42)	2.18 (−0.06)
8^2^CH_3_	C		1.36 (0.76)	1.86 (0.26)	−0.41 (2.53)
	D		1.10 (1.02)	1.67 (0.45)	−0.15 (2.27)
	CD	C	1.36 (0.76)	1.85 (0.27)	−0.44 (2.56)
		D	1.13 (0.99)	1.70 (0.42)	−0.12 (2.24)

**Table 2:** Predicted ^1^H chemical shifts and ring current shifts (in parentheses) of the heme substituents of domains C and D in the reduced state. The chemical shifts and ring current shifts were calculated from the crystal structure of bi-domain CD (PDB file 3OUE).

		Chemical Shifts (ppm)		
Heme Substituent	Domain	Heme I	Heme III	Heme IV
5H	C	9.45 (−0.09)	10.01 (−0.65)	9.39 (−0.03)
	D	9.47 (−0.11)	9.99 (−0.63)	9.36 (0.00)
10H	C	8.29 (1.07)	9.90 (−0.54)	9.59 (−0.23)
	D	8.44 (0.93)	9.71 (−0.35)	9.72 (−0.36)
15H	C	9.52 (−0.16)	9.81 (−0.45)	9.54 (−0.18)
	D	9.50 (−0.14)	9.67 (−0.31)	9.49 (−0.13)
20H	C	9.38 (−0.02)	9.85 (−0.49)	9.10 (0.26)
	D	9.42 (−0.06)	9.94 (−0.58)	9.28 (0.08)
2^1^CH_3_	C	3.44 (0.04)	4.34 (−0.86)	2.83 (0.66)
	D	3.60 (−0.12)	4.55 (−1.07)	3.42 (0.06)
7^1^CH_3_	C	3.44 (0.04)	3.97 (−0.49)	3.32 (0.16)
	D	3.37 (0.11)	3.93 (−0.45)	3.34 (0.14)
12^1^CH_3_	C	1.73 (1.75)	3.79 (−0.31)	2.81 (0.67)
	D	1.47 (2.01)	3.84 (−0.36)	3.42 (0.06)
18^1^CH_3_	C	3.51 (−0.03)	3.61 (−0.13)	3.42 (0.06)
	D	3.44 (0.04)	3.78 (−0.30)	3.49 (−0.01)
3^1^H	C	6.19 (−0.06)	6.72 (−0.59)	6.14 (−0.01)
	D	6.23 (−0.10)	6.67 (−0.54)	6.10 (0.03)
8^1^H	C	6.03 (0.10)	6.26 (−0.13)	6.40 (−0.27)
	D	5.97 (0.16)	6.05 (0.08)	6.56 (−0.43)
3^2^CH_3_	C	2.09 (0.03)	2.61 (−0.49)	2.03 (0.10)
	D	2.17 (−0.05)	2.43 (−0.31)	2.08 (0.04)
8^2^CH_3_	C	1.45 (0.67)	1.45 (0.67)	0.10 (2.02)
	D	0.95 (1.17)	1.62 (0.50)	0.53 (1.59)

## Data Availability

The data presented in this study are available on request from the corresponding author.

## References

[1] Lloyd J.R., Lovley D.R. (2001). Microbial detoxification of metals and radionuclides. Curr. Opin. Biotechnol.

[2] Holmes D.E., Finneran K.T., O’Neil R.A., Lovley D.R. (2002). Enrichment of members of the family *Geobacteraceae* associated with stimulation of dissimilatory metal reduction in uranium-contaminated aquifer sediments. Appl. Environ. Microbiol..

[3] Lovley D.R., Ueki T., Zhang T., Malvankar N.S., Shrestha P.M., Flanagan K.A., Aklujkar M., Butler J.E., Giloteaux L., Rotaru A.E. (2011). Geobacter: The microbe electric’s physiology, ecology, and practical applications. Adv. Microb. Physiol..

[4] Lovley D.R. (1991). Dissimilatory Fe(III) and Mn(IV) reduction. Microbiol. Rev..

[5] Lovley D.R. (1995). Bioremediation of organic and metal contaminants with dissimilatory metal reduction. J. Ind. Microbiol..

[6] Bond D.R., Lovley D.R. (2003). Electricity production by *Geobacter sulfurreducens* attached to electrodes. Appl. Environ. Microbiol..

[7] Malvankar N.S., Lovley D.R. (2014). Microbial nanowires for bioenergy applications. Curr. Opin. Biotechnol..

[8] Lovley D.R. (2011). Powering microbes with electricity: Direct electron transfer from electrodes to microbes. Environ. Microbiol. Rep..

[9] Wang L.Y., Nevin K.P., Woodard T.L., Mu B.Z., Lovley D.R. (2016). Expanding the Diet for DIET: Electron Donors Supporting Direct Interspecies Electron Transfer (DIET) in Defined Co-Cultures. Front. Microbiol..

[10] Lee K.Y., Bosch J., Meckenstock R.U. (2012). Use of metal-reducing bacteria for bioremediation of soil contaminated with mixed organic and inorganic pollutants. Environ. Geochem. Health.

[11] Salgueiro C.A., Morgado L., Silva M.A., Ferreira M.R., Fernandes T.M., Portela P.C. (2022). From iron to bacterial electroconductive filaments: Exploring cytochrome diversity using *Geobacter bacteria*. Coord. Chem. Rev..

[12] Wang F., Mustafa K., Suciu V., Joshi K., Chan C.H., Choi S., Su Z., Si D., Hochbaum A.I., Egelman E.H. (2022). Cryo-EM structure of an extracellular *Geobacter* OmcE cytochrome filament reveals tetrahaem packing. Nat. Microbiol..

[13] Wang F., Gu Y., O’Brien J.P., Yi S.M., Yalcin S.E., Srikanth V., Shen C., Vu D., Ing N.L., Hochbaum A.I. (2019). Structure of Microbial Nanowires Reveals Stacked Hemes that Transport Electrons over Micrometers. Cell.

[14] Wang F., Chan C.H., Suciu V., Mustafa K., Ammend M., Si D., Hochbaum A.I., Egelman E.H., Bond D.R. (2022). Structure of *Geobacter* OmcZ filaments suggests extracellular cytochrome polymers evolved independently multiple times. Elife.

[15] Pokkuluri P.R., Londer Y.Y., Duke N.E., Pessanha M., Yang X., Orshonsky V., Orshonsky L., Erickson J., Zagyanskiy Y., Salgueiro C.A. (2011). Structure of a novel dodecaheme cytochrome *c* from *Geobacter sulfurreducens* reveals an extended 12 nm protein with interacting hemes. J. Struct Biol..

[16] Pokkuluri P.R., Londer Y.Y., Duke N.E., Erickson J., Pessanha M., Salgueiro C.A., Schiffer M. (2004). Structure of a novel *c*_7_-type three-heme cytochrome domain from a multidomain cytochrome c polymer. Protein. Sci..

[17] Butler J.E., Young N.D., Lovley D.R. (2010). Evolution of electron transfer out of the cell: Comparative genomics of six *Geobacter genomes*. BMC Genom..

[18] Esteve-Nunez A., Sosnik J., Visconti P., Lovley D.R. (2008). Fluorescent properties of *c*-type cytochromes reveal their potential role as an extracytoplasmic electron sink in *Geobacter sulfurreducens*. Environ. Microbiol..

[19] Alves M.N., Fernandes A.P., Salgueiro C.A., Paquete C.M. (2016). Unraveling the electron transfer processes of a nanowire protein from *Geobacter sulfurreducens*. Biochim. Biophys. Acta.

[20] Page C.C., Moser C.C., Chen X., Dutton P.L. (1999). Natural engineering principles of electron tunnelling in biological oxidation-reduction. Nature.

[21] Morgado L., Paixao V.B., Schiffer M., Pokkuluri P.R., Bruix M., Salgueiro C.A. (2012). Revealing the structural origin of the redox-Bohr effect: The first solution structure of a cytochrome from *Geobacter sulfurreducens*. Biochem. J..

[22] Morgado L., Fernandes A.P., Londer Y.Y., Pokkuluri P.R., Schiffer M., Salgueiro C.A. (2009). Thermodynamic characterization of the redox centres in a representative domain of a novel *c*-type multihaem cytochrome. Biochem. J..

[23] Fernandes T.M., Morgado L., Turner D.L., Salgueiro C.A. (2021). Protein Engineering of Electron Transfer Components from Electroactive *Geobacter* Bacteria. Antioxidants.

[24] Fonseca B.M., Paquete C.M., Salgueiro C.A., Louro R.O. (2012). The role of intramolecular interactions in the functional control of multiheme cytochromes *c*. FEBS Lett..

[25] Santos H., Moura J.J., Moura I., LeGall J., Xavier A.V. (1984). NMR studies of electron transfer mechanisms in a protein with interacting redox centres: *Desulfovibrio gigas* cytochrome *c*_3_. Eur. J. Biochem..

[26] Salgueiro C.A., Turner D.L., Santos H., LeGall J., Xavier A.V. (1992). Assignment of the redox potentials to the four haems in *Desulfovibrio vulgaris* cytochrome *c*_3_ by 2D-NMR. FEBS Lett..

[27] Turner D.L., Salgueiro C.A., LeGall J., Xavier A.V. (1992). Structural studies of *Desulfovibrio vulgaris* ferrocytochrome *c*_3_ by two-dimensional NMR. Eur. J. Biochem..

[28] Turner D.L., Salgueiro C.A., Schenkels P., LeGall J., Xavier A.V. (1995). Carbon-^13^NMR studies of the influence of axial ligand orientation on haem electronic structure. Biochim. Biophys. Acta.

[29] Dantas J.M., Saraiva I.H., Morgado L., Silva M.A., Schiffer M., Salgueiro C.A., Louro R.O. (2011). Orientation of the axial ligands and magnetic properties of the hemes in the cytochrome *c*_7_ family from *Geobacter sulfurreducens* determined by paramagnetic NMR. Dalton. Trans..

[30] Morgado L., Fernandes A.P., Londer Y.Y., Bruix M., Salgueiro C.A. (2010). One simple step in the identification of the cofactors signals, one giant leap for the solution structure determination of multiheme proteins. Biochem. Biophys. Res. Commun..

[31] Keller R.M., Wuthrich K. (1978). Assignment of the heme *c* resonances in the 360 MHz H NMR spectra of cytochrome *c*. Biochim. Biophys. Acta.

[32] Qiu F., Rivera M., Stark R.E. (1998). An ^1^H-^13^C-^13^C-edited ^1^H NMR experiment for making resonance assignments in the active site of heme proteins. J. Magn. Reson..

[33] Ghini V., Chevance S., Turano P. (2019). About the use of ^(13)^C-^(13)^C NOESY in bioinorganic chemistry. J. Inorg. Biochem..

[34] Dantas J.M., Morgado L., Aklujkar M., Bruix M., Londer Y.Y., Schiffer M., Pokkuluri P.R., Salgueiro C.A. (2015). Rational engineering of *Geobacter sulfurreducens* electron transfer components: A foundation for building improved *Geobacter*-based bioelectrochemical technologies. Front. Microbiol..

[35] McDonald C.C., Phillips W.D., Vinogradov S.N. (1969). Proton magnetic resonance evidence for methionine-iron coordination in mammalian-type ferrocytochrome *c*. Biochem. Biophys. Res. Commun..

[36] Moore G.R., Pettigrew G.W. (1990). Cytochromes C: Evolutionary, Structural and Physicochemical Aspects.

[37] Pessanha M., Brennan L., Xavier A.V., Cuthbertson P.M., Reid G.A., Chapman S.K., Turner D.L., Salgueiro C.A. (2001). NMR structure of the haem core of a novel tetrahaem cytochrome isolated from *Shewanella frigidimarina*: Identification of the haem-specific axial ligands and order of oxidation. FEBS Lett..

[38] Pessanha M., Londer Y.Y., Long W.C., Erickson J., Pokkuluri P.R., Schiffer M., Salgueiro C.A. (2004). Redox characterization of *Geobacter sulfurreducens* cytochrome *c*_7_: Physiological relevance of the conserved residue F15 probed by site-specific mutagenesis. Biochemistry.

[39] Harada E., Fukuoka Y., Ohmura T., Fukunishi A., Kawai G., Fujiwara T., Akutsu H. (2002). Redox-coupled conformational alternations in cytochrome *c*_3_ from *D. vulgaris* Miyazaki F on the basis of its reduced solution structure. J. Mol. Biol..

[40] Harada E., Kumagai J., Ozawa K., Imabayashi S., Tsapin A.S., Nealson K.H., Meyer T.E., Cusanovich M.A., Akutsu H. (2002). A directional electron transfer regulator based on heme-chain architecture in the small tetraheme cytochrome *c* from *Shewanella oneidensis*. FEBS Lett..

[41] Louro R.O., Pacheco I., Turner D.L., LeGall J., Xavier A.V. (1996). Structural and functional characterization of cytochrome *c*_3_ from *D. desulfuricans* ATCC 27774 by ^1^H-NMR. FEBS Lett..

[42] Picarra-Pereira M.A., Turner D.L., LeGall J., Xavier A.V. (1993). Structural studies on *Desulfovibrio gigas* cytochrome *c*_3_ by two-dimensional ^1^H-nuclear-magnetic-resonance spectroscopy. Biochem. J..

[43] Coutinho I.B., Turner D.L., LeGall J., Xavier A.V. (1993). Characterization of the structure and redox behaviour of cytochrome *c*_3_ from *Desulfovibrio baculatus* by ^1^H-nuclear-magnetic-resonance spectroscopy. Biochem. J..

[44] Pereira P.M., Pacheco I., Turner D.L., Louro R.O. (2002). Structure-function relationship in type II cytochrome *c*_3_ from *Desulfovibrio africanus*: A novel function in a familiar heme core. J. Biol. Inorg. Chem..

[45] Morgado L., Bruix M., Orshonsky V., Londer Y.Y., Duke N.E., Yang X., Pokkuluri P.R., Schiffer M., Salgueiro C.A. (2008). Structural insights into the modulation of the redox properties of two *Geobacter sulfurreducens* homologous triheme cytochromes. Biochim. Biophys. Acta.

[46] Moss G.P. (1988). Nomenclature of tetrapyrroles. Recommendations 1986 IUPAC-IUB Joint Commission on Biochemical Nomenclature (JCBN). Eur. J. Biochem..

[47] Londer Y.Y., Pokkuluri P.R., Orshonsky V., Orshonsky L., Schiffer M. (2006). Heterologous expression of dodecaheme “nanowire” cytochromes *c* from *Geobacter sulfurreducens*. Protein. Expr. Purif..

[48] Londer Y.Y., Pokkuluri P.R., Erickson J., Orshonsky V., Schiffer M. (2005). Heterologous expression of hexaheme fragments of a multidomain cytochrome from *Geobacter sulfurreducens* representing a novel class of cytochromes *c*. Protein. Expr. Purif..

[49] Fernandes A.P., Nunes T.C., Paquete C.M., Salgueiro C.A. (2017). Interaction studies between periplasmic cytochromes provide insights into extracellular electron transfer pathways of *Geobacter sulfurreducens*. Biochem. J..

[50] Gordon E.H., Steensma E., Ferguson S.J. (2001). The cytochrome *c* domain of dimeric cytochrome *cd*_1_ of *Paracoccus pantotrophus* can be produced at high levels as a monomeric holoprotein using an improved *c*-type cytochrome expression system in *Escherichia coli*. Biochem. Biophys. Res. Commun..

[51] Arslan E., Schulz H., Zufferey R., Kunzler P., Thony-Meyer L. (1998). Overproduction of the *Bradyrhizobium japonicum c*-type cytochrome subunits of the cbb3 oxidase in *Escherichia coli*. Biochem. Biophys. Res. Commun..

[52] Seeliger S., Cord-Ruwisch R., Schink B. (1998). A periplasmic and extracellular *c*-type cytochrome of *Geobacter sulfurreducens* acts as a ferric iron reductase and as an electron carrier to other acceptors or to partner bacteria. J. Bacteriol..

[53] Pierattelli R., Banci L., Turner D.L. (1996). Indirect determination of magnetic susceptibility tensors in peroxidases: A novel approach to structure elucidation by NMR. J. Biol. Inorg. Chem..

[54] Pessanha M., Rothery E.L., Miles C.S., Reid G.A., Chapman S.K., Louro R.O., Turner D.L., Salgueiro C.A., Xavier A.V. (2009). Tuning of functional heme reduction potentials in *Shewanella fumarate* reductases. Biochim. Biophys. Acta.

